# A machine learning approach for predicting 72-hour mortality of hypothermic patients only using non-invasive parameters: A multi-center retrospective cohort study

**DOI:** 10.1371/journal.pone.0334526

**Published:** 2025-10-22

**Authors:** Chunliang Jiang, GuoFeng Ru, GuanJun Liu, Huiquan Wang, Xin Ma, JiaMeng Xu, YiJing Fu, Jing Yuan, Guang Zhang

**Affiliations:** 1 School of Control Science and Engineering, Tiangong University, Tianjin, China; 2 Systems Engineering Institute, Academy of Military Sciences, People’s Liberation Army, Tianjin, China; 3 Tianjin Key Laboratory for Advanced Mechatronic System Design and Intelligent Control, School of Mechanical Engineering, Tianjin University of Technology, Tianjin, China; University of Washington, UNITED STATES OF AMERICA

## Abstract

**Objectives:**

Accurately predicting the mortality risk of hypothermia patients is crucial for clinical decision-making, offering ample time for physicians to intervene. However, existing methods are invasive and difficult to implement in pre-hospital settings.

**Methods:**

In this study, records of 2,700 hypothermia patients from 125 hospitals were extracted from the eICU Collaborative Research database, comprising 360 non-survivors and 2,340 survivors. Four machine learning methods were utilized to develop a mortality prediction model for hypothermia patients based on non-invasive physiological parameters. Data from 122 hospitals were used for model training, while the remainder were utilized for performance validation.

**Results:**

The proposed machine learning prediction model achieved an area under the receiver operating characteristic curve (AUC) of 0.869 (95%CI: 0.840–0.895), demonstrating good mortality predictive performance for hypothermia patients only using non-invasive physiological parameters. Optimal and minimal feature subsets were selected for each machine learning method. The optimal feature subsets contained only 70.6% of the overall features for XGBoost models, while the AUC values increased by 0.039 compared to that of the entire feature subset. The interpretability analysis results highlight the vital importance of the temperature feature in predicting mortality during episodes of hypothermia in patients.

**Conclusions:**

This study developed a mortality prediction method for hypothermia patients only using non-invasive parameters, demonstrating robustness and reliability during multi-center validation. It can offer decision support for remote areas and disaster sites where it is difficult to access invasive parameters.

## 1. Introduction

Hypothermia, whether resulting from environmental exposure, illness, or injury, remains a global clinical challenge and is closely associated with increased early mortality in critically ill patients. It affects multiple physiological systems and complicates critical care by exacerbating coagulopathy, acidosis, and tissue hypoperfusion [1–5]. Patients with varying degrees of hypothermia display distinct symptoms, but once they reach a state of severe hypothermia, irreversible complications arise, which can also lead to death.

Hypothermia is commonly defined as the condition of a core temperature ≤35°C [6–9]. Hypothermia is frequently observed among critically ill patients and is an independent predictor of early mortality. Hsieh et al. demonstrated that emergency department hypothermia significantly increases mortality risk in trauma populations [10–11]. Therefore, early mortality prediction for patients with hypothermia appears particularly crucial.

Extensive research on the association between hypothermia and mortality risk has been conducted. The team of Ralf Erkens et al. [10] and Ting-Min Hsieh et al. [11] found that the assessment of core body temperature upon admission to the ICU is a predictive factor for patient mortality rates. However, relying solely on core body temperature as a predictive factor is insufficient. Kirsten Balvers et al. [8] conducted a retrospective study on hypothermic trauma patients and reported a correlation between mortality and physiological parameters. In contrast, Mathieu Pasquier et al. [12] conducted a study in Switzerland focusing on vital signs and physiological characteristics in accidental hypothermia. A patient mortality prediction model for risk assessment after admission was also developed. Statistical methods and scoring systems have been used to analyze the correlation between the mortality rates of hypothermic patients and factors such as the type of injury, Glasgow Coma Scale (GCS), age, climate, physical condition, as well as invasive parameters like pH and creatinine [13–15]. Statistical methods and traditional scoring systems rely on predefined scoring metrics and criteria, demanding high data quality from patient records. Moreover, these models are overly simplistic, failing to fully account for the complexity and diversity of actual situations [2,7–9,15].

In addition to exploring factors associated with mortality due to hypothermia, some scholars have developed and validated machine learning models utilizing physiological data to predict the mortality of patients with hypothermia. Yohei Okada et al. [17] used physiological data obtained during hospital admission from six hospitals, comparing three machine-learning models with two traditional scoring models. The top-performing random forest model had an AUC value of 0.794, while SOFA (Sequential Organ Failure Assessment Score) and 5A (Age, Activities of daily living, Arrest or hemodynamically unstable, Acidemia, and Albumin) score method achieved 0.787 and 0.750, respectively [7]. These findings demonstrate that machine learning models can predict in-hospital mortality rates in patients with accidental hypothermia, and outperform traditional scoring models. Nevertheless, these predictive models require a substantial number of vital signs and laboratory data, such as albumin and serum potassium [1,7,16–18]. Additionally, the lack of specialized medical laboratory equipment in remote rural areas and disaster-stricken regions limits the obtaining of invasive and laboratory physiological parameters, limiting the widespread application of these models in remote areas.

In general, previous studies have exhibited certain limitations. Firstly, simple statistical methods fail to fully consider the complexity and diversity of real situations. Secondly, traditional scoring systems and early mortality prediction methods relying on invasive and laboratory physiological parameters are not suitable for remote areas. Thirdly, the previous studies are single-center retrospective cohort studies that have yet to undergo further validation through large-scale, multicenter clinical trials, leaving their general applicability in clinical practice uncertain. The above factors have limited the widespread application of these methods in clinical practice [19].

To enhance the precision of prediction results and fulfill the broad application needs in both pre-hospital and in-hospital care, this study developed four machine learning prediction models solely utilizing non-invasive physiological parameters. The performance of these models was trained using data from 122 hospitals and validated with data from 3 additional hospitals to enhance the representativeness and generalizability of the study results.

## 2. Methods

### 2.1. Definitions

**Hypothermia** Hypothermia is defined as two consecutive core body temperature records below 35°C within a span of at least 10 minutes.

**Observation window**: The time interval with available data used to predict the mortality of hypothermic patients.

**Prediction window** During this time interval, an assessment is conducted to determine the mortality of patients with hypothermia.

**Prediction gap** The time interval between the observation window and the prediction window, which is set to 1 hour. Prediction gap is designated for advanced medical interventions, aiming at reducing the risk of complications and mortality.

**Fig 1:**
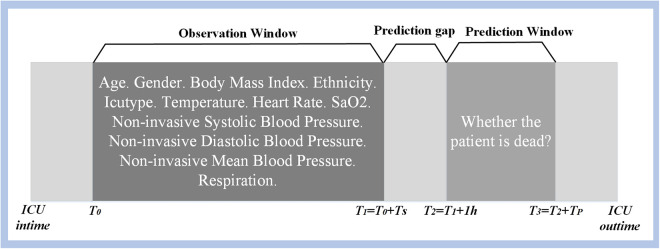
Definitions of mortality prediction for patients with hypothermia.

The definition of mortality prediction for hypothermic patients is illustrated in Fig 1. Throughout the modeling phase, patient physiological data was used for both training and testing purposes. Specifically, information gathered within the observation window$T_{S}$ ([*T*_*0*_*, T*_*1*_]) following ICU admission was employed for training the model aimed at predicting patients’ mortality risk within the $T_{P}$ [*T*_*2*_*, T*_*3*_]) time interval.

### 2.2. Overall process of mortality prediction in hypothermic patients

**Fig 2:**
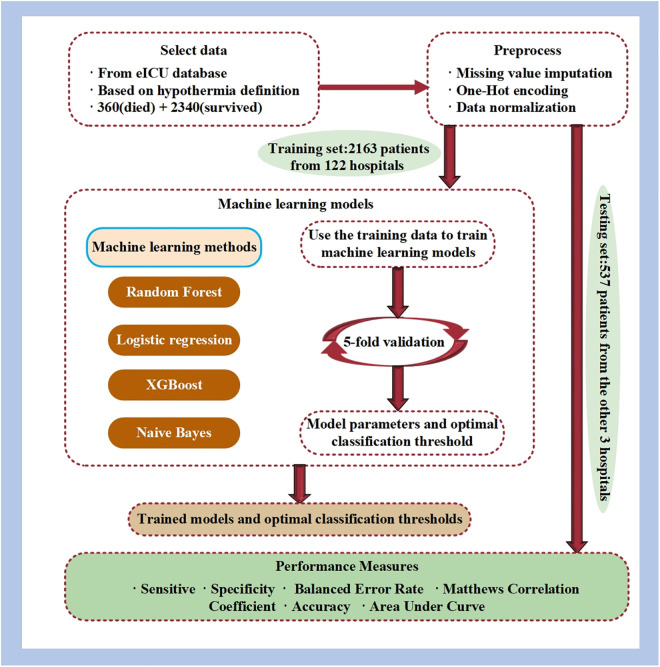
Overall process of mortality prediction in patients with hypothermia.

The process of mortality prediction in patients with hypothermia is shown in Fig 2.

1) Data was obtained from the eICU Collaborative Research Database (eICU) according to the hypothermia definition.
2) Missing data underwent imputation employing interpolation rules aligned with clinical practice guidelines before standardization.
3) Cross-validation was employed during model training to acquire models featuring robust and optimal hyperparameters.
4) The test set was used to evaluate the predictive performance of four machine learning methods.

### 2.3. Study population

#### 2.3.1. Data source.

The data used in this study was sourced from the eICU database, which aggregates ICU data from over 400 hospitals in the United States, comprising anonymized health data from over 200,000 ICU inpatients. The eICU database is a valuable resource for clinical research, housing an extensive range of high-quality clinical information encompassing vital signs, diagnoses, and treatments [20]. This study is a retrospective analysis based on a database and did not involve any clinical trials. The data used in this study were extracted from the “eICU” database (https://physionet.org/content/eicu-crd/2.0/), which is a publicly available dataset. The use of these databases complies with the ethical standards for research involving human data. No patient identifiers were used, ensuring the confidentiality and privacy of the individuals represented in the data.

#### 2.3.2. Enrollment criteria.

To target the early warning of mortality risk in hypothermia patients, available samples were selected from the database. To eliminate factors that could potentially affect the modeling outcomes, this study imposed several enrollment criterias during data selection, as follows:

a) Patients age should be between 18 and 65 years old;
b) Only patients who developed hypothermia after ICU admission defined as two consecutive core body temperature measurements below 35°C were included in the study;
c) After the occurrence of hypothermia, the time series for all seven key physiological parameters should be longer than 4 hours.

All hypothermic patients meeting the inclusion criteria were considered, regardless of whether the etiology was traumatic or non-traumatic. This approach was chosen to reflect real-world clinical situations in which the cause of hypothermia may not be readily known at the time of early assessment.

**Fig 3:**
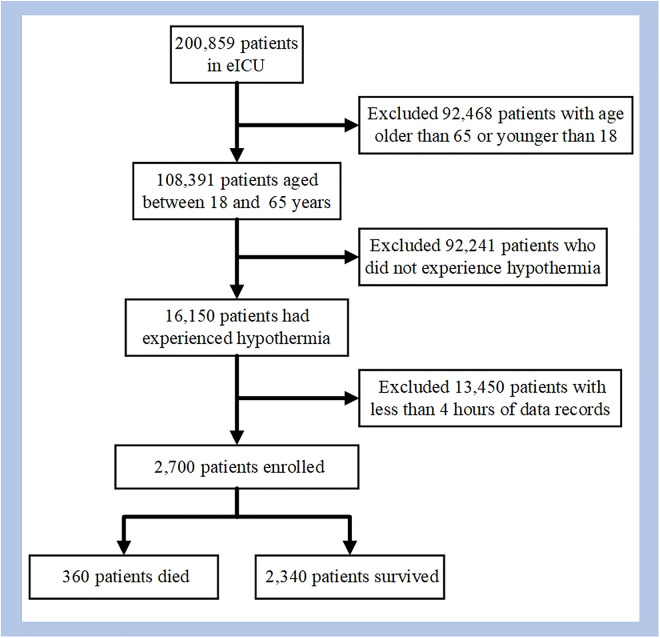
Process for selection of the study cohort.

The specific process of data screening is shown in Fig 3.

### 2.4. Parameter selection and construction of feature matrix

To extract more valuable information from the raw data records for model construction, this study employed 12 non-invasive physiological parameters listed in Table 1 to develop a mortality prediction model for patients with hypothermia. For seven physiological parameters, the maximum value, minimum value, mean, variance, standard deviation, skewness, upper quartile, median, and lower quartile were computed individually, resulting in a total of 68 features including five demographic parameters, to construct the feature matrix. The definitions for all feature values of seven physiological parameters are described as follows:

**Table 1:** Non-invasive physiological parameters included in the study.

Subset	Detail
Demographic parameters	Age, BMI, Gender, Ethnicity, Icutype
Physiological parameters	Temperature, Heart Rate, Respiration, SpO2, Non-invasive Systolic Blood pressure, Non-invasive Diastolic Blood pressure, Non-invasive Mean Blood pressure

The maximum value of each physiological parameter indicates the largest value of the physiological parameter recorded in the observation window, and the minimum value and the average value have the same meaning.

The variance is defined as the statistical measure that quantifies the dispersion between data points and the mean. The calculation formula is shown in Eq. (1).


$$s^{2}=\frac{\text{1}}{n}\sum_{i=1}^{n}(x_{i}-\overset{―}{x}{)}^{2}$$ (1)

The standard deviation is defined as the square root of the variance, representing the degree of dispersion in the data. The calculation formula is shown in Eq. (2).


$$s=\sqrt{\frac{\text{1}}{n}\sum_{i=1}^{n}{\left(x_{i}-\overset{―}{x}\right)}^{2}}$$ (2)

Skewness is defined as the statistical measure that reflects the degree of asymmetry in the distribution of data. The calculation formula is shown in Eq. (3).


$$Sk=\frac{\frac{\text{1}}{n}\sum {\mathrm{\backslash nolimits}}_{i=1}^{n}{\left(x_{i}-\overset{―}{x}\right)}^{3}}{{\left(\sqrt{\frac{\text{1}}{n}\sum {\mathrm{\backslash nolimits}}_{i=1}^{n}{\left(x_{i}-\overset{―}{x}\right)}^{2}}\right)}^{3}}$$ (3)

The lower quartile is defined as the value at the 25th percentile of the data when arranged in ascending order. The median is defined as the value at the 50th percentile. The upper quartile is defined as the value at the 75th percentile.

### 2.5. Data pre-processing

Original Electronic Health Records (EHRs) often contain a plethora of noise, primarily due to artifacts from patient movement, equipment malfunction, and other factors resulting in outliers and missing values. Direct application of these data would hinder the construction of accurate and reliable predictive models [21]. Therefore, preprocessing the data before use is an essential step.

**Outlier Treatment** In this study, a combination of physicians’ clinical experience and statistical methods was employed to identify outliers. Initially, based on clinical judgment, all physiological parameters should be positive, and data with body temperatures below 25°C or above 45°C, heart rates exceeding 250 bpm, respiratory rates over 70 bpm, and blood pressure values above 300 mmHg were considered abnormal. To enhance the precision of outlier detection, the 3-sigma (3σ) rule was applied to the dataset, and records falling outside the mean ± 3 standard deviations of the physiological parameters were deemed outliers. Identified outliers were subsequently removed, and their original positions in the dataset were marked as empty values.

**Missing Value Imputation** For the missing and anomalous values in the original dataset, interpolation procedures will be implemented. The specific rules for imputation are as follows: Missing data will be addressed through linear interpolation based on the records immediately preceding and following the missing value. In the absence of adjacent records, nearest-neighbor imputation will be employed. Specifically for missing non-invasive blood pressure data, invasive blood pressure measurements from the same time period will be used for imputation. The missing values for the remaining statistical parameters and body mass index (BMI) were imputed using mean values.

**One-Hot Encoding** One-hot encoding converts categorical variables into binary vectors where each bit represents a category [22]. In this study, gender, ethnicity, ICU type, and patient survival status were processed using one-hot encoding.

**Normalization** Normalization is a data preprocessing technique used to scale numerical features to a similar range, often between 0 and 1 or −1 and 1 [23]. In this study, to eliminate the influence of dimensional disparities among different features, all data were subjected to normalization to ensure that their values fall within the range of 0 and 1.

### 2.6. Assessment of feature value importance

The study utilized a combined selection method to evaluate the impact of features on the model through the sorting of their importance [24,25]. The filtering methods comprised ReliefF [26], Fisher criterion [27], and Gini index [28] were used to calculate the feature importance. These three algorithms evaluate the importance of features from multiple perspectives. The final weight is derived from the summation of the normalized results of these three filtering methods, as is illustrated in Eq. (4), with higher weights indicating more crucial feature variables.


$$IW=ReliefF_{norm}+Fisher_{norm}-Gini_{norm}$$ (4)

Where IW is the importance weight of each feature, ReliefF_norm_, Fisher_norm_ and Gini_norm_ are the normalization value of ReliefF, Fisher and Gini index for each feature.

### 2.7. Feature selection

For broader applicability and to avoid excessive reliance on processing capacity, determining feature subsets is paramount importance. In this research, feature selection aims to identify the optimal feature subset (OPT_subset) that achieves the best model performance, and the minimal feature subset (MIN_subset) that ensures acceptable performance with the fewest features of non-invasive physiological parameters. Adding features to the input set of machine learning methods based on their importance ranking, computing the average balanced error rate (BER) and standard error for each machine learning method. The number of features corresponding to the point with the minimum average BER was selected as the number of features in the OPT_subset. Likewise, the number of features corresponding to the point where the mean BER was within one standard error of the lowest BER was considered as the number of features in the MIN_subset [29].

### 2.8. Prediction method

In this study, four traditional machine learning methods were utilized to predict the mortality of hypothermic patients based on non-invasive physiological parameters. These methods comprised Random Forest [30], Logistic Regression [31], Naive Bayes [32] and Extreme Gradient Boosting [33], which are commonly used classical machine learning algorithms The optimal preset parameters for each method were derived through Bayesian debugging.

### 2.9. Performance evaluation

#### 2.9.1. Training and Test Sets.

**Fig 4:**
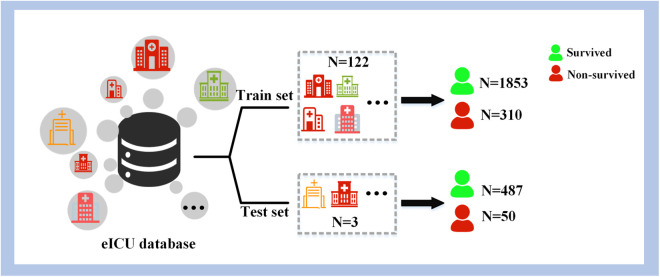
Partitioning into training and testing sets.

The data was divided into training and testing sets, as illustrated in Fig 4. The training set, comprising 2,163 patients from 122 hospitals, was utilized to determine the model’s optimal parameters via cross-validation and to ascertain the best classification threshold in conjunction with cost-sensitive theory. The test set included 537 patients from 3 hospitals was solely used for performance evaluation. Employing a multicenter data approach for testing the model’s generalizability.

#### 2.9.2. Performance measures.

Sensitivity, Specificity, Balanced Error Rate, Matthews Correlation Coefficient (MCC), Accuracy and Area Under Curve (AUC) were hereby used as performance evaluation criteria [33]. Table 2 displays the utilized formulas along with concise descriptions of each metric.

**Table 2:** Metrics used to evaluate the prediction performance.

Formula	Description
$SEN=\frac{TP}{TP+FN}$	The proportion of true positive instances correctly predicted by the algorithm model.
$SPE=\frac{TN}{TN+FP}$	The proportion of negative points that are correctly predicted by the algorithm model as negative points.
$BER=\frac{\text{1}}{\text{2}}*\left(\frac{FP}{FP+TN}+\frac{FN}{FN+TP}\right)$	The lowest error rate that the classifier can achieve on a data set.
$MCC=\frac{TP\cdot TN-FP\cdot FN}{\sqrt{\left(TP+FP\right)+\left(TP+FN\right)+\left(TN+FP\right)+\left(TN+FN\right)}}$	Describe the correlation coefficient between the actual classification and the predicted classification.
$BER=\frac{\text{1}}{\text{2}}*\left(\frac{FP}{FP+TN}+\frac{FN}{FN+TP}\right)$	Accuracy classification score.
$AUC=\frac{\sum {\mathrm{\backslash nolimits}}_{d=1}^{D}\left(TPR_{\left(d\right)}+TPR_{\left(d+1\right)}\right)\cdot \left(FPR_{\left(d\right)}+FPR_{\left(d+1\right)}\right)}{\text{2}}$	Used to measure the classifier’s ability to differentiate between positive and negative samples, where higher values indicate better model performance.

## 3. Results

### 3.1. Baseline characteristics of included patients

Based on the inclusion criteria outlined in Section 2.3.2, this study selected 2700 patients from the eICU database. Among them, after the occurrence of hypothermia, 360 patients (13.33% of the total) did not survive, while 2,340 patients survived. The baseline characteristics of the included patients are shown in Table 3 Compared to the surviving group, patients in the non-survived group exhibited a higher average age (51.62 ± 11.69 years vs. 50.55 ± 11.89 years, p < 0.05) and higher BMI values (37.98 ± 17.92 vs. 30.46 ± 16.85, p < 0.001), as well as significant differences in parameters such as body temperature, heart rate, and blood pressure between the two groups.

**Table 3:** Baseline characteristics of included patients.

Variable	All patients	Survived	Non- Survived	P value
	(*N* = 2700)	(*N* = 2340)	(*N* = 360)	
Age (year), mean ± std	50.75 ± 11.86	50.55 ± 11.89	51.62 ± 11.69	P < 0.05
Male sex, n (%)	1592 (58.96)	1280 (54.70)	312 (86.67)	0.342
BMI, mean ± std	31.87 ± 17.62	30.46 ± 16.85	37.98 ± 17.92	P < 0.001
Temperature, mean ± std	35.24 ± 1.91	35.36 ± 1.93	34.43 ± 1.55	P < 0.001
Heart Rate, mean ± std	86.75 ± 19.81	85.95 ± 19.60	92.01 ± 20.44	P < 0.001
Respiration, mean ± std	19.78 ± 6.04	19.53 ± 5.91	21.34 ± 6.58	P < 0.001
SaO2, mean ± std	95.37 ± 5.82	95.92 ± 4.42	91.77 ± 10.60	P < 0.001
Non-invasive Systolic, mean ± std	116.99 ± 21.60	118.30 ± 21.27	108.48 ± 21.81	P < 0.001
Non-invasive Diastolic, mean ± std	65.22 ± 13.62	65.69 ± 13.32	62.17 ± 15.21	P < 0.001
Non-invasive Mean, mean ± std	81.12 ± 15.40	81.80 ± 15.04	76.66 ± 16.91	P < 0.001
Type of ICU, n (%)				P < 0.05
Cardiac ICU	165 (6.11)	121 (5.53)	44 (8.58)	
CCU-CTICU	224 (8.30)	188 (8.60)	36 (7.02)	
CSICU	15 (0.56)	11 (0.50)	4 (0.78)	
CTICU	107 (3.96)	95 (4.34)	12 (2.34)	
Med-Surg ICU	1472 (54.52)	1171 (53.54)	301 (58.67)	
MICU	297 (11)	244 (11.16)	53 (10.33)	
Neuro ICU	179 (6.63)	152 (6.95)	27 (5.26)	
SICU	241 (8.93)	205 (9.37)	36 (7.02)	
Ethnicity, n, (%)				0.167
Hispanic	132 (4.89)	115 (4.91)	17 (4.72)	
Native American	22 (0.81)	15 (0.64)	7 (1.94)	
Asian	62 (2.30)	53 (2.26)	9 (2.50)	
Caucasian	1935 (71.67)	1682 (71.88)	253 (70.28)	
African American	384 (14.22)	334 (14.27)	50 (13.89)	
Other	165 (6.11)	141 (4.87)	24 (6.67)	

### 3.2. Prediction performance with different prediction windows and observation windows

In an effort to provide timely alerts for potential risks and accurate prediction, this study analyzed and evaluated the impact of various observation window (i.e., 1, 2, 3, and 4 hours) and prediction windows (i.e., 24, 36, 48, 60, and 72 hours) on model performance. Overall, a total of 20 different window combinations were obtained to validate the predictive performance of each prediction algorithm.

**Fig 5:**
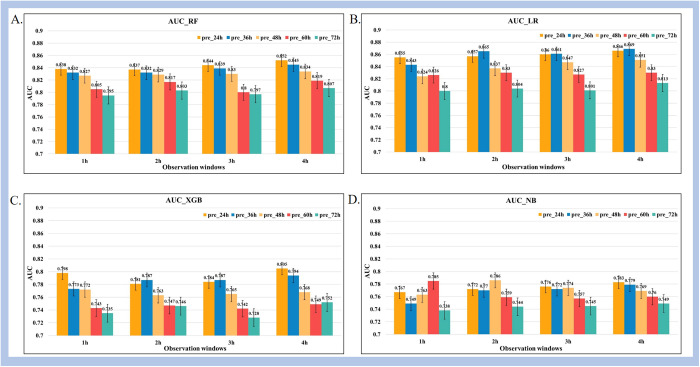
Trends in AUC values for different observation windows and prediction windows for (A) Random Forest, (B) Logistic Regression, (C) XGBoost, and (D) Naïve Bayes four machine learning methods. Observation window length is set to 1h, 2h, 3h and 4h, respectively; Pre_n: prediction window with length *n* hours.

**Fig 6:**
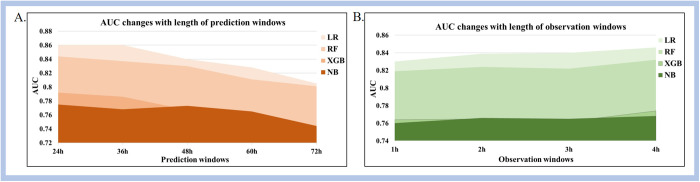
Trends for average AUC for (A) different prediction window and (B) different observation window for each model.

Fig 5 and 6 illustrates the variations in AUC values under diverse observation and prediction windows after conducting cross-validation across multiple hospitals. Detailed results are illustrated in S1 Appendix The results show that, on one hand, under the same prediction window, the model’s AUC value improves with an increase in the observation window length. On the other hand, under the same observation window, the model’s AUC value decreases as the prediction window length increases.

Among various combinations of observation and prediction windows, the AUC values of the four machine learning methods were consistently above 0.728. The LR model achieved the largest AUC value of 0.869 (95%CI: 0.840–0.895) when the observation window was set to 4 hours and the prediction window to 36 hours. It indicates that all the machine learning methods exhibit relatively strong predictive performance in mortality prediction of hypothermic patients only utilizing non-invasive parameters.

### 3.3. Effect of feature reduction on the performance of prediction algorithms

By calculating the mean and standard error of BER, the MIN_subset and OPT_subset were determined and compared with the predictive performance of the model using all features with the observation window of 4 hours and the prediction window of 24 hours.

**Fig 7:**
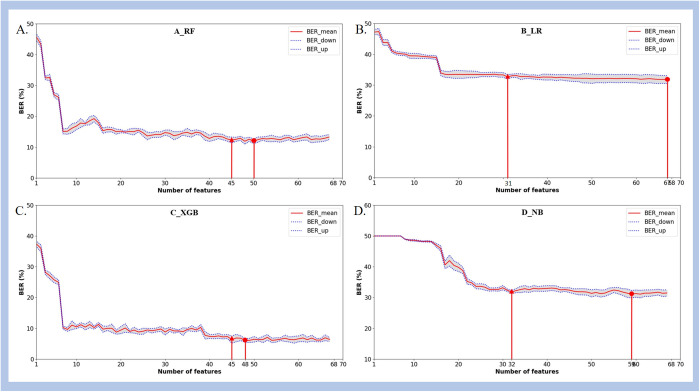
Feature selection results for (A) Random Forest, (B) Logistic Regression, (C) XGBoost, and (D) Naïve Bayes. The central line represents the mean, while the gray area denotes one standard error range. Triangles and dots, along with their associated numbers, signify the MIN_subset and OPT_subset, respectively. **A)** The BER of random forest, **B)** The BER of logistic regression, **C)** The BER of XGBoost, **D)** The BER of Naive Bayes.

Fig 7 shows the trend of BER variation as features are added to each model one by one, from the feature with the highest weight to the one with the lowest weight, according to the results of feature selection for four machine learning methods. Table 4 presents the predictive performance of various prediction models under corresponding feature subsets. Among all predictive models, the MIN_subset contained no more than 45 features, representing a reduction of over 33.8%, with a maximum decrease of 4.6% in AUC value. The LR model observed a reduction of 54.4% in the number of features within the MIN_subset, while the AUC value decreased by only 1.5%, resulting in an AUC of 0.853 (95%CI: 0.817–0.886). The number of feature variables in the OPT_subset ranges from 48 to 67. Compared with the prediction performance of all features, the XGBoost model using optimal subset obtained the largest reduction of 29.4% in number of features, while its AUC values increased by 3.7%, resulting in an AUC of 0.835 (95%CI: 0.802–0.869).

**Table 4:** Identification results of four algorithms on test sets for different feature subsets.

Feature Select	Methods	Num	Result					
			ACC (95%CI)	AUC (95%CI)	BER (95%CI)	MCC (95%CI)	SEN (95%CI)	SPE (95%CI)
MIN_subset	RF	45(66.2%)	0.782 (0.768-0.797)	0.854 (0.820-0.886)	0.222 (0.189-0.257)	0.273 (0.234-0.312)	0.783 (0.768-0.797)	0.773 (0.706-0.839)
	LR	31(45.6%)	0.797 (0.783-0.811)	0.853 (0.817-0.886)	0.202 (0.170-0.234)	0.298 (0.261-0.336)	0.797 (0.783-0.811)	0.800 (0.736-0.861)
	XGB	45(66.2%)	0.746 (0.731-0.761)	0.810 (0.773-0.846)	0.260 (0.223-0.295)	0.226 (0.190-0.264)	0.747 (0.731-0.762)	0.733 (0.664-0.804)
	NB	32(47.1%)	0.690 (0.674-0.707)	0.747 (0.705-0.788)	0.315 (0.277-0.354)	0.167 (0.130-0.202)	0.691 (0.675-0.707)	0.680 (0.604-0.754)
OPT_subset	RF	50(73.5%)	0.782 (0.768-0.797)	0.855 (0.822-0.826)	0.221 (0.188-0.257)	0.270 (0.231-0.307)	0.783 (0.768-0.798)	0.767 (0.698-0.831)
	LR	67(98.5%)	0.812 (0.799-0.826)	0.866 (0.831-0.897)	0.190 (0.158-0.225)	0.317 (0.275-0.357)	0.812 (0.799-0.826)	0.807 (0.741-0.870)
	XGB	48(70.6%)	0.776 (0.761-0.790)	0.835 (0.802-0.869)	0.229 (0.195-0.263)	0.264 (0.226-0.302)	0.776 (0.762-0.791)	0.767 (0.701-0.834)
	NB	59(86.8%)	0.723 (0.707-0.738)	0.783 (0.739-0.816)	0.280 (0.243-0.318)	0.203 (0.165-0.240)	0.723 (0.707-0.739)	0.717 (0.642-0.788)
ALL Features	RF	68(100%)	0.787 (0.752-0.781)	0.852 (0.816-0.884)	0.227 (0.194-0.260)	0.263 (0.224-0.299)	0.786 (0.751-0.781)	0.780 (0.714-0.845)
	LR	68(100%)	0.816 (0.803-0.830)	0.866 (0.832-0.897)	0.191 (0.160-0.225)	0.318 (0.277-0.357)	0.817 (0.803-0.831)	0.800 (0.733-0.860)
	XGB	68(100%)	0.737 (0.721-0.751)	0.805 (0.768-0.840)	0.271 (0.235-0.307)	0.214 (0.176-0.250)	0.738 (0.722-0.752)	0.720 (0.648-0.788)
	NB	68(100%)	0.729 (0.713-0.744)	0.783 (0.743-0.821)	0.280 (0.243-0.318)	0.204 (0.165-0.241)	0.729 (0.714-0.745)	0.710 (0.635-0.783)

### 3.4. Feature importance evaluation

**Fig 8:**
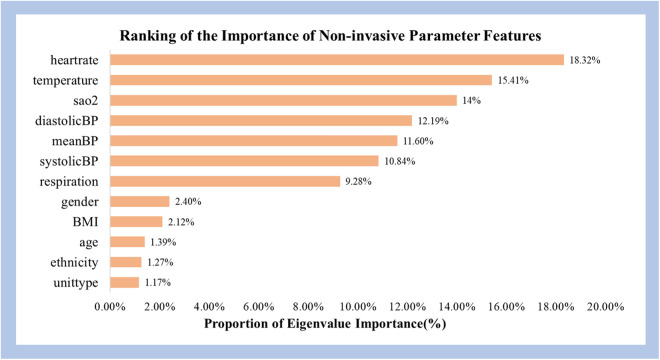
Proportion of Feature Importance.

Fig 8. illustrates the proportion of feature importance scores for different non-invasive physiological parameters. In research predicting the mortality risk of hypothermia patients, the feature importance scores of three non-invasive physiological parameters—heart rate, body temperature, and blood oxygen saturation—were the most significant, collectively accounting for 47.73%. Upon further inclusion of non-invasive systolic blood pressure, non-invasive diastolic blood pressure, and non-invasive mean arterial pressure, the total contribution increased to 82.36%. This weight distribution highlights the significant role of heartrate, temperature, blood oxygen saturation and blood pressure in predicting the mortality risk of hypothermia patients.

## 4. Discussion

The rapid advancement of artificial intelligence has made patient mortality prediction more accurate [34–38]. However, the widespread application of existing prediction models for hypothermia patient mortality is limited in remote areas. To predict mortality among these patients, we develop a prediction method that only uses non-invasive physiological parameters. Four machine learning models were employed to build the prediction model and validated with data across multiple centers. The findings demonstrate that these machine learning methods achieve relatively high predictive performance. Additionally, feature reduction was utilized to reduce computational costs, and interpretability analysis was conducted to assess the significance of features in predicting mortality among hypothermia patients.

This study assessed the impact of different observation and prediction windows on predictive performance. The findings revealed that an increase in the observation window length resulted in an overall enhancement of the model’s predictive performance. This improvement is attributed to the longer observation window encompassing more comprehensive information on the evolution of patient conditions. Conversely, under the same observation window, extending the prediction window length led to a decline in the model’s predictive performance due to increased noise and interference. This further substantiates the significance of selecting appropriate combinations of observation and prediction window lengths for accurate mortality prediction of hypothermic patients.

By ranking the importance of features from different categories of non-invasive physiological parameters, the study explores the impact of various parameters on the predictive performance for mortality risk due to hypothermia. Key non-invasive physiological parameters such as heart rate, body temperature, blood oxygen saturation and blood pressure hold substantial weight in the model’s predictions, accounting for 82.36% of the total. These physiological parameters can sensitively reflect the changes in the respiratory and circulatory systems of hypothermic patients and are frequently used in common death risk scores such as the SOFA score, MODS score, and SIRS score.

Feature selection were conducted to reduce feature dimensionality and enhance computational efficiency while maintaining high predictive performance. Compared to using all features, the LR model’s minimal feature subset constituted only 45.6% of the complete features, resulting in a mere 0.013 decrease in its AUC value. Based on the optimal feature subsets, the AUC values of RF and XGBoost models exhibited an increase of 0.003 and 0.03, respectively. This demonstrates that even with a reduced number of features, the model maintains robust predictive performance while reducing computational costs, thereby enhancing the applicability of predictive models.

This study conducted multi-center validation of the model’s performance, with AUC values consistently exceeding 0.728 and reaching a maximum of 0.869. These results indicate the high reliability and broad applicability of the predictive model across diverse environments, populations, and conditions.

This study has also some limitations. First, the data used in this study is based on the eICU database, which might contain errors and noise in information recording. Future efforts should focus on developing denoising algorithms to implement these methods in clinical settings, enhancing the model’s stability and reliability. Second, the distinction between traumatic and non-traumatic hypothermia was not made, which may affect etiological specificity despite improving model robustness and generalizability. Third, static observation windows were employed in this study, potentially failing to capture the dynamic trends in patient data over time. Hence, future research will explore more adaptable dynamic observation windows to better suit timely prediction requirements. Finally, the core focus of this study is to explore the feasibility of machine learning methods in predicting mortality among hypothermic patients. Future endeavors will involve experimenting with ensemble learning and deep learning techniques to enhance the predictive performance and accuracy of the model, providing reliable support for clinical decision-making.

## 5. Conclusions

In summary, a prediction model for hypothermia mortality was developed and validated in this study, which used only non-invasive physiological parameters in a large data set. The predictive models also demonstrated excellent performance when validated in multiple clinical centers. In the clinical setting, the non-invasive methods outlined in this study offer a more efficient means to promptly identify patients at high risk of hypothermia, enabling earlier implementation of necessary therapeutic interventions. This study is expected to improve the treatment success rate and survival rate among hypothermia patients in remote and disaster areas.

## Supporting information

S1 Appendix Appendix A: Detailed results are illustrated. (DOCX)

S1 Data Appendix DATA: Patient dataset. (ZIP)
