# Supplementary material for: A machine learning approach for predicting 72-hour mortality of hypothermic patients only using non-invasive parameters: A multi-center retrospective cohort study
Source: PLoS One. 2025 Oct 22;20(10):e0334526. doi: 10.1371/journal.pone.0334526 (PMC12543198; doi:10.1371/journal.pone.0334526)
Supplement: S1 Appendix — (DOCX) [file pone.0334526.s001.docx]

**Table 1 The prediction performance values of four machine learning methods under different observation windows and prediction windows using only non-invasive parameters.**

| **Methods** | **Time** | **ACC (95%CI)** | **AUC (95%CI)** | **BER (95%CI)** | **MCC (95%CI)** | **SEN (95%CI)** | **SPE (95%CI)** |
| --- | --- | --- | --- | --- | --- | --- | --- |
|  | Win_1h-Pre_24h | 0.772  (0.744-0.800) | 0.838  (0.784-0.901) | 0.225  (0.160-0.297) | 0.261  (0.181-0.332) | 0.771  (0.743-0.800) | 0.778  (0.636-0.903) |
|  | Win_1h-Pre_36h | 0.758  (0.727-0.787) | 0.832  (0.786-0.892) | 0.239  (0.179-0.305) | 0.273  (0.196-0.344) | 0.758  (0.726-0.788) | 0.763  (0.636-0.880) |
| RF | Win_1h-Pre_48h | 0.759  (0.729-0.787) | 0.827  (0.781-0.878) | 0.239  (0.182-0.301) | 0.288  (0.214-0.360) | 0.759  (0.728-0.788) | 0.762  (0.640-0.875) |
|  | Win_1h-Pre_60h | 0.730  (0.698-0.759) | 0.805  (0.761-0.854) | 0.272  (0.219-0.329) | 0.276  (0.203-0.346) | 0.730  (0.698-0.760) | 0.725  (0.617-0.827) |
|  | Win_1h-Pre_72h | 0.730  (0.698-0.760) | 0.795  (0.738-0.847) | 0.269  (0.219-0.321) | 0.290  (0.218-0.357) | 0.730  (0.696-0.761) | 0.733  (0.630-0.830) |
|  | Win_1h-Pre_24h | 0.803  (0.776-0.831) | 0.855  (0.802-0.917) | 0.196  (0.131-0.268) | 0.302  (0.218-0.375) | 0.803  (0.775-0.831) | 0.806  (0.658-0.930) |
|  | Win_1h-Pre_36h | 0.793  (0.768-0.823) | 0.843  (0.808-0.912) | 0.199  (0.143-0.259) | 0.329  (0.252-0.402) | 0.796  (0.766-0.824) | 0.806  (0.686-0.914) |
| LR | Win_1h-Pre_48h | 0.811  (0.783-0.837) | 0.824  (0.793-0.898) | 0.190  (0.138-0.248) | 0.364  (0.285-0.438) | 0.811  (0.782-0.838) | 0.810  (0.698-0.911) |
|  | Win_1h-Pre_60h | 0.757  (0.728-0.788) | 0.826  (0.782-0.874) | 0.248  (0.196-0.304) | 0.313  (0.238-0.384) | 0.758  (0.727-0.790) | 0.746  (0.638-0.848) |
|  | Win_1h-Pre_72h | 0.720  (0.689-0.750) | 0.800  (0.745-0.853) | 0.277  (0.225-0.330) | 0.277  (0.206-0.348) | 0.719  (0.688-0.752) | 0.727  (0.625-0.826) |
|  | Win_1h-Pre_24h | 0.762  (0.722-0.792) | 0.798  (0.756-0.882) | 0.244  (0.173-0.320) | 0.242  (0.163-0.318) | 0.763  (0.722-0.793) | 0.750  (0.600-0.886) |

**Table 1 (continued)**

| **Methods** | **Time** | **ACC (95%CI)** | **AUC (95%CI)** | **BER (95%CI)** | **MCC (95%CI)** | **SEN (95%CI)** | **SPE (95%CI)** |
| --- | --- | --- | --- | --- | --- | --- | --- |
|  | Win_1h-Pre_36h | 0.699  (0.667-0.739) | 0.773  (0.702-0.843) | 0.296  (0.230-0.366) | 0.203  (0.129-0.275) | 0.698  (0.665-0.739) | 0.710  (0.571-0.837) |
| XGB | Win_1h-Pre_48h | 0.704  (0.672-0.735) | 0.772  (0.723-0.836) | 0.296  (0.236-0.360) | 0.218  (0.144-0.282) | 0.704  (0.671-0.736) | 0.705  (0.578-0.820) |
|  | Win_1h-Pre_60h | 0.652  (0.619-0.684) | 0.743  (0.680-0.799) | 0.345  (0.288-0.404) | 0.179  (0.108-0.245) | 0.651  (0.616-0.684) | 0.659  (0.544-0.767) |
|  | Win_1h-Pre_72h | 0.679  (0.646-0.710) | 0.735  (0.695-0.809) | 0.323  (0.269-0.381) | 0.215  (0.141-0.284) | 0.680  (0.645-0.712) | 0.673  (0.563-0.777) |
|  | Win_1h-Pre_24h | 0.721  (0.691-0.754) | 0.767  (0.693-0.866) | 0.285  (0.210-0.366) | 0.194  (0.116-0.268) | 0.722  (0.691-0.754) | 0.708  (0.550-0.852) |
|  | Win_1h-Pre_36h | 0.734  (0.702-0.763) | 0.749  (0.704-0.855) | 0.267  (0.201-0.336) | 0.238  (0.161-0.311) | 0.734  (0.701-0.765) | 0.731  (0.600-0.860) |
| NB | Win_1h-Pre_48h | 0.744  (0.714-0.775) | 0.763  (0.745-0.853) | 0.261  (0.201-0.325) | 0.261  (0.187-0.333) | 0.745  (0.714-0.776) | 0.733  (0.610-0.850) |
|  | Win_1h-Pre_60h | 0.731  (0.701-0.761) | 0.785  (0.732-0.834) | 0.272  (0.218-0.328) | 0.277  (0.203-0.348) | 0.732  (0.700-0.764) | 0.725  (0.616-0.829) |
|  | Win_1h-Pre_72h | 0.711  (0.681-0.741) | 0.738  (0.695-0.808) | 0.291  (0.238-0.347) | 0.259  (0.187-0.330) | 0.712  (0.679-0.744) | 0.707  (0.600-0.810) |
|  | Win_2h-Pre_24h | 0.757  (0.736-0.777) | 0.837  (0.803-0.889) | 0.235  (0.192-0.280) | 0.277  (0.224-0.328) | 0.756  (0.734-0.777) | 0.764  (0.687-0.859) |
|  | Win_2h-Pre_36h | 0.742  (0.721-0.763) | 0.832  (0.784-0.874) | 0.250  (0.250-0.298) | 0.257  (0.204-0.307) | 0.741  (0.719-0.763) | 0.758  (0.667-0.842) |
| RF | Win_2h-Pre_48h | 0.778  (0.758-0.798) | 0.829  (0.813-0.883) | 0.220  (0.179-0.261) | 0.319  (0.267-0.373) | 0.778  (0.757-0.798) | 0.782  (0.702-0.860) |

**Table 1 (continued)**

| **Methods** | **Time** | **ACC (95%CI)** | **AUC (95%CI)** | **BER (95%CI)** | **MCC (95%CI)** | **SEN (95%CI)** | **SPE (95%CI)** |
| --- | --- | --- | --- | --- | --- | --- | --- |
|  | Win_2h-Pre_60h | 0.745  (0.723-0.765) | 0.817  (0.787-0.852) | 0.252  (0.216-0.291) | 0.306  (0.254-0.354) | 0.744  (0.721-0.766) | 0.752  (0.677-0.820) |
|  | Win_2h-Pre_72h | 0.734  (0.713-0.755) | 0.803  (0.764-0.840) | 0.272  (0.235-0.310) | 0.287  (0.236-0.335) | 0.736  (0.712-0.758) | 0.720  (0.645-0.790) |
|  | Win_2h-Pre_24h | 0.812  (0.792-0.831) | 0.857  (0.815-0.911) | 0.191  (0.145-0.240) | 0.311  (0.252-0.366) | 0.812  (0.792-0.831) | 0.806  (0.710-0.893) |
|  | Win_2h-Pre_36h | 0.799  (0.779-0.818) | 0.865  (0.821-0.905) | 0.198  (0.157-0.241) | 0.332  (0.275-0.384) | 0.798  (0.778-0.818) | 0.806  (0.720-0.885) |
| LR | Win_2h-Pre_48h | 0.802  (0.783-0.821) | 0.837  (0.805-0.885) | 0.199  (0.161-0.238) | 0.354  (0.299-0.406) | 0.802  (0.782-0.822) | 0.801  (0.723-0.875) |
|  | Win_2h-Pre_60h | 0.764  (0.744-0.786) | 0.830  (0.789-0.867) | 0.236  (0.200-0.275) | 0.332  (0.279-0.382) | 0.765  (0.743-0.786) | 0.762  (0.689-0.831) |
|  | Win_2h-Pre_72h | 0.729  (0.708-0.750) | 0.804  (0.762-0.842) | 0.272  (0.236-0.310) | 0.286  (0.235-0.335) | 0.729  (0.707-0.752) | 0.727  (0.652-0.796) |
|  | Win_2h-Pre_24h | 0.714  (0.691-0.736) | 0.781  (0.721-0.838) | 0.289  (0.237-0.344) | 0.189  (0.136-0.238) | 0.714  (0.692-0.736) | 0.708  (0.600-0.808) |
|  | Win_2h-Pre_36h | 0.696  (0.674-0.718) | 0.787  (0.733-0.837) | 0.300  (0.254-0.350) | 0.199  (0.147-0.247) | 0.696  (0.673-0.718) | 0.704  (0.611-0.794) |
| XGB | Win_2h-Pre_48h | 0.709  (0.686-0.731) | 0.763  (0.719-0.823) | 0.294  (0.249-0.340) | 0.221  (0.168-0.273) | 0.709  (0.686-0.732) | 0.704  (0.613-0.790) |
|  | Win_2h-Pre_60h | 0.663  (0.641-0.686) | 0.747  (0.702-0.790) | 0.337  (0.295-0.377) | 0.192  (0.141-0.242) | 0.663  (0.639-0.688) | 0.663  (0.584-0.742) |
|  | Win_2h-Pre_72h | 0.682  (0.659-0.704) | 0.746  (0.702-0.786) | 0.324  (0.285-0.363) | 0.215  (0.165-0.263) | 0.683  (0.658-0.707) | 0.670  (0.592-0.745) |

**Table 1 (continued)**

| **Methods** | **Time** | **ACC (95%CI)** | **AUC (95%CI)** | **BER (95%CI)** | **MCC (95%CI)** | **SEN (95%CI)** | **SPE (95%CI)** |
| --- | --- | --- | --- | --- | --- | --- | --- |
|  | Win_2h-Pre_24h | 0.734  (0.712-0.755) | 0.772  (0.724-0.835) | 0.268  (0.217-0.321) | 0.212  (0.159-0.262) | 0.734  (0.712-0.755) | 0.729  (0.625-0.830) |
|  | Win_2h-Pre_36h | 0.721  (0.699-0.741) | 0.770  (0.720-0.816) | 0.282  (0.234-0.330) | 0.222  (0.168-0.272) | 0.722  (0.699-0.743) | 0.715  (0.621-0.807) |
| NB | Win_2h-Pre_48h | 0.732  (0.710-0.752) | 0.786  (0.743-0.826) | 0.273  (0.230-0.316) | 0.248  (0.196-0.298) | 0.732  (0.710-0.755) | 0.722  (0.637-0.806) |
|  | Win_2h-Pre_60h | 0.710  (0.688-0.732) | 0.759  (0.719-0.798) | 0.287  (0.249-0.326) | 0.256  (0.205-0.307) | 0.710  (0.686-0.733) | 0.716  (0.640-0.788) |
|  | Win_2h-Pre_72h | 0.689  (0.666-0.711) | 0.744  (0.705-0.781) | 0.310  (0.271-0.350) | 0.232  (0.180-0.281) | 0.689  (0.666-0.712) | 0.690  (0.613-0.763) |
|  | Win_3h-Pre_24h | 0.782  (0.766-0.798) | 0.844  (0.797-0.886) | 0.222  (0.182-0.262) | 0.268  (0.222-0.311) | 0.783  (0.766-0.799) | 0.773  (0.691-0.849) |
|  | Win_3h-Pre_36h | 0.766  (0.749-0.782) | 0.839  (0.800-0.875) | 0.240  (0.204-0.279) | 0.275  (0.231-0.318) | 0.767  (0.749-0.784) | 0.753  (0.676-0.824) |
| RF | Win_3h-Pre_48h | 0.765  (0.748-0.781) | 0.830  (0.807-0.875) | 0.234  (0.201-0.268) | 0.303  (0.260-0.345) | 0.765  (0.748-0.782) | 0.766  (0.701-0.829) |
|  | Win_3h-Pre_60h | 0.723  (0.705-0.740) | 0.800  (0.766-0.832) | 0.279  (0.247-0.311) | 0.269  (0.227-0.310) | 0.724  (0.705-0.742) | 0.719  (0.656-0.778) |
|  | Win_3h-Pre_72h | 0.730  (0.712-0.748) | 0.797  (0.763-0.829) | 0.270  (0.239-0.300) | 0.288  (0.246-0.329) | 0.730  (0.711-0.749) | 0.729  (0.671-0.788) |
|  | Win_3h-Pre_24h | 0.797  (0.781-0.813) | 0.860  (0.819-0.897) | 0.203  (0.166-0.243) | 0.292  (0.245-0.336) | 0.797  (0.781-0.813) | 0.796  (0.718-0.869) |
|  | Win_3h-Pre_36h | 0.793  (0.777-0.810) | 0.861  (0.826-0.894) | 0.212  (0.178-0.248) | 0.315  (0.271-0.359) | 0.794  (0.777-0.811) | 0.781  (0.711-0.849) |

**Table 1 (continued)**

| **Methods** | **Time** | **ACC (95%CI)** | **AUC (95%CI)** | **BER (95%CI)** | **MCC (95%CI)** | **SEN (95%CI)** | **SPE (95%CI)** |
| --- | --- | --- | --- | --- | --- | --- | --- |
| LR | Win_3h-Pre_48h | 0.770  (0.753-0.787) | 0.847  (0.825-0.878) | 0.228  (0.195-0.262) | 0.312  (0.268-0.352) | 0.770  (0.753-0.787) | 0.775  (0.709-0.838) |
|  | Win_3h-Pre_60h | 0.756  (0.738-0.772) | 0.827  (0.795-0.856) | 0.242  (0.213-0.271) | 0.321  (0.281-0.361) | 0.755  (0.737-0.772) | 0.761  (0.703-0.815) |
|  | Win_3h-Pre_72h | 0.724  (0.701-0.746) | 0.801  (0.761-0.840) | 0.279  (0.242-0.318) | 0.276  (0.226-0.326) | 0.725  (0.702-0.747) | 0.717  (0.642-0.786) |
|  | Win_3h-Pre_24h | 0.700  (0.682-0.717) | 0.784  (0.729-0.814) | 0.296  (0.253-0.341) | 0.181  (0.137-0.221) | 0.700  (0.681-0.717) | 0.708  (0.621-0.791) |
|  | Win_3h-Pre_36h | 0.724  (0.706-0.742) | 0.787  (0.743-0.828) | 0.278  (0.240-0.316) | 0.226  (0.183-0.268) | 0.724  (0.706-0.742) | 0.720  (0.644-0.793) |
| XGB | Win_3h-Pre_48h | 0.699  (0.681-0.718) | 0.765  (0.726-0.806) | 0.302  (0.265-0.337) | 0.214  (0.173-0.256) | 0.699  (0.680-0.719) | 0.697  (0.628-0.768) |
|  | Win_3h-Pre_60h | 0.663  (0.644-0.681) | 0.742  (0.693-0.771) | 0.342  (0.308-0.375) | 0.186  (0.145-0.227) | 0.664  (0.644-0.684) | 0.652  (0.587-0.716) |
|  | Win_3h-Pre_72h | 0.671  (0.652-0.689) | 0.728  (0.692-0.764) | 0.332  (0.299-0.363) | 0.203  (0.164-0.244) | 0.671  (0.651-0.690) | 0.664  (0.606-0.725) |
|  | Win_3h-Pre_24h | 0.730  (0.713-0.747) | 0.776  (0.741-0.828) | 0.267  (0.226-0.311) | 0.212  (0.168-0.252) | 0.730  (0.712-0.748) | 0.736  (0.649-0.816) |
|  | Win_3h-Pre_36h | 0.717  (0.699-0.735) | 0.772  (0.733-0.809) | 0.283  (0.245-0.322) | 0.219  (0.176-0.260) | 0.717  (0.698-0.735) | 0.717  (0.639-0.792) |
| NB | Win_3h-Pre_48h | 0.722  (0.704-0.740) | 0.774  (0.739-0.808) | 0.280  (0.245-0.317) | 0.242  (0.198-0.281) | 0.722  (0.704-0.741) | 0.718  (0.647-0.784) |
|  | Win_3h-Pre_60h | 0.701  (0.683-0.719) | 0.757  (0.725-0.789) | 0.298  (0.266-0.331) | 0.242  (0.200-0.282) | 0.701  (0.683-0.720) | 0.702  (0.639-0.763) |

**Table 1 (continued)**

| **Methods** | **Time** | **ACC (95%CI)** | **AUC (95%CI)** | **BER (95%CI)** | **MCC (95%CI)** | **SEN (95%CI)** | **SPE (95%CI)** |
| --- | --- | --- | --- | --- | --- | --- | --- |
|  | Win_3h-Pre_72h | 0.699  (0.680-0.717) | 0.745  (0.713-0.775) | 0.304  (0.271-0.335) | 0.241  (0.200-0.283) | 0.700  (0.680-0.718) | 0.693  (0.633-0.754) |
|  | Win_4h-Pre_24h | 0.787  (0.752-0.781) | 0.852  (0.816-0.884) | 0.227  (0.194-0.260) | 0.263  (0.224-0.299) | 0.786  (0.751-0.781) | 0.780  (0.714-0.845) |
|  | Win_4h-Pre_36h | 0.768  (0.753-0.782) | 0.845  (0.813-0.875) | 0.228  (0.199-0.258) | 0.291  (0.255-0.326) | 0.767  (0.752-0.782) | 0.776  (0.717-0.833) |
| RF | Win_4h-Pre_48h | 0.743  (0.728-0.757) | 0.834  (0.796-0.854) | 0.253  (0.225-0.281) | 0.282  (0.246-0.317) | 0.742  (0.727-0.757) | 0.752  (0.695-0.806) |
|  | Win_4h-Pre_60h | 0.741  (0.725-0.756) | 0.819  (0.791-0.847) | 0.264  (0.237-0.291) | 0.294  (0.257-0.239) | 0.742  (0.726-0.758) | 0.731  (0.677-0.781) |
|  | Win_4h-Pre_72h | 0.726  (0.711-0.741) | 0.807  (0.775-0.830) | 0.274  (0.248-0.301) | 0.283  (0.247-0.317) | 0.727  (0.710-0.742) | 0.725  (0.673-0.775) |
|  | Win_4h-Pre_24h | 0.816  (0.803-0.830) | 0.866  (0.832-0.897) | 0.191  (0.160-0.225) | 0.318  (0.277-0.357) | 0.817  (0.803-0.831) | 0.800  (0.733-0.860) |
|  | Win_4h-Pre_36h | 0.798  (0.785-0.812) | 0.869  (0.840-0.895) | 0.206  (0.177-0.236) | 0.328  (0.288-0.364) | 0.799  (0.785-0.813) | 0.789  (0.729-0.846) |
| LR | Win_4h-Pre_48h | 0.766  (0.751-0.780) | 0.851  (0.821-0.878) | 0.241  (0.213-0.270) | 0.302  (0.263-0.339) | 0.767  (0.752-0.783) | 0.750  (0.693-0.805) |
|  | Win_4h-Pre_60h | 0.751  (0.736-0.765) | 0.830  (0.800-0.852) | 0.249  (0.223-0.276) | 0.315  (0.278-0.349) | 0.751  (0.735-0.766) | 0.752  (0.701-0.800) |
|  | Win_4h-Pre_72h | 0.725  (0.709-0.739) | 0.813  (0.785-0.838) | 0.270  (0.244-0.296) | 0.287  (0.251-0.322) | 0.724  (0.707-0.739) | 0.737  (0.686-0.787) |
|  | Win_4h-Pre_24h | 0.737  (0.721-0.751) | 0.805  (0.768-0.840) | 0.271  (0.235-0.307) | 0.214  (0.176-0.250) | 0.738  (0.722-0.752) | 0.720  (0.648-0.788) |

**Table 1 (continued)**

| **Methods** | **Time** | **ACC (95%CI)** | **AUC (95%CI)** | **BER (95%CI)** | **MCC (95%CI)** | **SEN (95%CI)** | **SPE (95%CI)** |
| --- | --- | --- | --- | --- | --- | --- | --- |
|  | Win_4h-Pre_36h | 0.723  (0.708-0.738) | 0.794  (0.756-0.829) | 0.276  (0.244-0.309) | 0.230  (0.193-0.266) | 0.723  (0.707-0.739) | 0.724  (0.659-0.787) |
| XGB | Win_4h-Pre_48h | 0.706  (0.691-0.721) | 0.768  (0.723-0.796) | 0.297  (0.267-0.327) | 0.225  (0.188-0.260) | 0.707  (0.691-0.723) | 0.699  (0.640-0.755) |
|  | Win_4h-Pre_60h | 0.695  (0.679-0.710) | 0.749  (0.715-0.770) | 0.313  (0.285-0.342) | 0.226  (0.189-0.260) | 0.697  (0.680-0.713) | 0.697  (0.623-0.730) |
|  | Win_4h-Pre_72h | 0.697  (0.681-0.711) | 0.752  (0.723-0.779) | 0.306  (0.279-0.333) | 0.239  (0.203-0.272) | 0.697  (0.681-0.713) | 0.692  (0.638-0.742) |
|  | Win_4h-Pre_24h | 0.729  (0.713-0.744) | 0.783  (0.743-0.821) | 0.280  (0.243-0.318) | 0.204  (0.165-0.241) | 0.729  (0.714-0.745) | 0.710  (0.635-0.783) |
|  | Win_4h-Pre_36h | 0.712  (0.696-0.727) | 0.779  (0.734-0.802) | 0.285  (0.252-0.319) | 0.220  (0.183-0.255) | 0.712  (0.696-0.727) | 0.719  (0.651-0.781) |
| NB | Win_4h-Pre_48h | 0.707  (0.691-0.722) | 0.769  (0.728-0.799) | 0.294  (0.264-0.324) | 0.229  (0.193-0.263) | 0.707  (0.691-0.723) | 0.705  (0.646-0.763) |
|  | Win_4h-Pre_60h | 0.708  (0.693-0.723) | 0.760  (0.729-0.784) | 0.299  (0.272-0.326) | 0.245  (0.210-0.280) | 0.709  (0.693-0.725) | 0.693  (0.640-0.745) |
|  | Win_4h-Pre_72h | 0.749  (0.680-0.712) | 0.749  (0.722-0.776) | 0.304  (0.277-0.331) | 0.240  (0.205-0.275) | 0.696  (0.680-0.713) | 0.695  (0.643-0.748) |

Win_n: observation window length; Pre_n: prediction window length.

**Table 2 The prediction performance values of four machine learning methods with invasive parameters.**

| **Methods** | **Time** | **ACC (95%CI)** | **AUC (95%CI)** | **BER (95%CI)** | **MCC (95%CI)** | **SEN (95%CI)** | **SPE (95%CI)** |
| --- | --- | --- | --- | --- | --- | --- | --- |
|  | Win_4h-Pre_24h | 0.815  (0.802-0.829) | 0.881  (0.849-0.908) | 0.198  (0.166-0.233) | 0.311  (0.269-0.351) | 0.817  (0.803-0.830) | 0.787  (0.719-0.850) |
|  | Win_4h-Pre_36h | 0.815  (0.802-0.828) | 0.881  (0.853-0.907) | 0.180  (0.153-0.207) | 0.363  (0.326-0.400) | 0.814  (0.801-0.827) | 0.826  (0.771-0.878) |
| RF | Win_4h-Pre_48h | 0.803  (0.789-0.817) | 0.866  (0.840-0.890) | 0.194  (0.169-0.221) | 0.371  (0.332-0.406) | 0.802  (0.788-0.817) | 0.810  (0.758-0.858) |
|  | Win_4h-Pre_60h | 0.786  (0.772-0.800) | 0.864  (0.840-0.885) | 0.209  (0.186-0.235) | 0.375  (0.338-0.410) | 0.784  (0.770-0.799) | 0.797  (0.748-0.841) |
|  | Win_4h-Pre_72h | 0.773  (0.758-0.787) | 0.850  (0.826-0.874) | 0.224  (0.199-0.249) | 0.358  (0.322-0.394) | 0.772  (0.757-0.787) | 0.780  (0.732-0.826) |
|  | Win_4h-Pre_24h | 0.766  (0.751-0.780) | 0.810  (0.764-0.853) | 0.231  (0.198-0.265) | 0.259  (0.220-0.296) | 0.765  (0.750-0.780) | 0.773  (0.705-0.838) |
|  | Win_4h-Pre_36h | 0.779  (0.764-0.793) | 0.829  (0.790-0.865) | 0.220  (0.192-0.251) | 0.304  (0.265-0.314) | 0.778  (0.763-0.793) | 0.781  (0.720-0.837) |
| LR | Win_4h-Pre_48h | 0.731  (0.715-0.746) | 0.767  (0.725-0.805) | 0.278  (0.248-0.309) | 0.251  (0.214-0.288) | 0.732  (0.716-0.748) | 0.712  (0.652-0.751) |
|  | Win_4h-Pre_60h | 0.691  (0.675-0.707) | 0.749  (0.713-0.785) | 0.306  (0.278-0.334) | 0.233  (0.197-0.268) | 0.690  (0.673-0.707) | 0.698  (0.644-0.752) |
|  | Win_4h-Pre_72h | 0.692  (0.676-0.708) | 0.736  (0.700-0.772) | 0.311  (0.282-0.340) | 0.231  (0.195-0.268) | 0.693  (0.676-0.710) | 0.685  (0.631-0.740) |
|  | Win_4h-Pre_24h | 0.759  (0.744-0.773) | 0.826  (0.787-0.862) | 0.249  (0.214-0.285) | 0.240  (0.202-0.278) | 0.759  (0.745-0.774) | 0.743  (0.674-0.811) |
|  | Win_4h-Pre_36h | 0.762  (0.747-0.777) | 0.804  (0.772-0.836) | 0.246  (0.220-0.273) | 0.327  (0.291-0.365) | 0.764  (0.748-0.779) | 0.743  (0.694-0.794) |

**Table 2 (continued)**

| **Methods** | **Time** | **ACC (95%CI)** | **AUC (95%CI)** | **BER (95%CI)** | **MCC (95%CI)** | **SEN (95%CI)** | **SPE (95%CI)** |
| --- | --- | --- | --- | --- | --- | --- | --- |
| XGB | Win_4h-Pre_48h | 0.719  (0.704-0.734) | 0.784  (0.746-0.821) | 0.275  (0.247-0.305) | 0.252  (0.215-0.286) | 0.718  (0.702-0.734) | 0.731  (0.674-0.786) |
|  | Win_4h-Pre_60h | 0.736  (0.720-0.751) | 0.782  (0.749-0.814) | 0.265  (0.239-0.293) | 0.291  (0.254-0.326) | 0.737  (0.720-0.752) | 0.733  (0.680-0.783) |
|  | Win_4h-Pre_72h | 0.724  (0.708-0.739) | 0.762  (0.716-0.804) | 0.279  (0.247-0.313) | 0.228  (0.191-0.264) | 0.724  (0.708-0.740) | 0.719  (0.654-0.782) |
|  | Win_4h-Pre_24h | 0.825  (0.811-0.838) | 0.875  (0.845-0.902) | 0.182  (0.151-0.216) | 0.332  (0.289-0.373) | 0.825  (0.812-0.839) | 0.810  (0.744-0.869) |
|  | Win_4h-Pre_36h | 0.810  (0.796-0.823) | 0.874  (0.850-0.896) | 0.193  (0.163-0.221) | 0.348  (0.309-0.389) | 0.810  (0.796-0.824) | 0.805  (0.747-0.862) |
| NB | Win_4h-Pre_48h | 0.784  (0.770-0.799) | 0.828  (0.796-0.858) | 0.221  (0.193-0.250) | 0.332  (0.294-0.369) | 0.785  (0.770-0.800) | 0.774  (0.718-0.827) |
|  | Win_4h-Pre_60h | 0.772  (0.757-0.786) | 0.827  (0.801-0.853) | 0.231  (0.207-0.258) | 0.342  (0.306-0.380) | 0.772  (0.757-0.788) | 0.764  (0.715-0.813) |
|  | Win_4h-Pre_72h | 0.761  (0.746-0.777) | 0.804  (0.774-0.834) | 0.235  (0.210-0.261) | 0.340  (0.303-0.376) | 0.760  (0.745-0.776) | 0.770  (0.720-0.817) |

Win_n: observation window length; Pre_n: prediction window length.
